# WALTZ-DB 2.0: an updated database containing structural information of experimentally determined amyloid-forming peptides

**DOI:** 10.1093/nar/gkz758

**Published:** 2019-09-02

**Authors:** Nikolaos Louros, Katerina Konstantoulea, Matthias De Vleeschouwer, Meine Ramakers, Joost Schymkowitz, Frederic Rousseau

**Affiliations:** 1 VIB Center for Brain & Disease Research, Switch Laboratory, Leuven, 3000, Belgium; 2 KU Leuven, Department of Cellular and Molecular Medicine, Leuven, 3000, Belgium

## Abstract

Transition of soluble proteins into insoluble amyloid fibrils is driven by self-propagating short sequence stretches. However, accurate prediction of aggregation determinants remains challenging. Here, we describe WALTZ-DB 2.0, an updated and significantly expanded open-access database providing information on experimentally determined amyloid-forming hexapeptide sequences (http://waltzdb.switchlab.org/). We have updated WALTZ-DB 2.0 with new entries, including: (i) experimental validation of an in-house developed dataset of 229 hexapeptides, using electron microscopy and Thioflavin-T binding assays; (ii) manual curation of 98 amyloid-forming peptides isolated from literature. Furthermore, the content has been expanded by adding novel structural information for peptide entries, including sequences of the previous version. Using a computational methodology developed in the Switch lab, we have generated 3D-models of the putative amyloid fibril cores of WALTZ-DB 2.0 entries. Structural models, coupled with information on the energetic contributions and fibril core stabilities, can be accessed through individual peptide entries. Customized filtering options for subset selections and new modelling graphical features were added to upgrade online accessibility, providing a user-friendly interface for browsing, downloading and updating. WALTZ-DB 2.0 remains the largest open-access repository for amyloid fibril formation determinants and will continue to enhance the development of new approaches focused on accurate prediction of aggregation prone sequences.

## INTRODUCTION

Protein folding is a crucial process during which polypeptide chains adopt a thermodynamically stable three-dimensional structure that is pivotal for most cellular functions. Proteins that misfold or fail to retain their native tertiary structure are prone to forming amyloid fibril aggregates (1). Amyloids are linked to a growing number of widespread debilitating diseases, including type II diabetes (T2D), atherosclerosis, systemic amyloidoses and capital neurodegenerative diseases, such as Alzheimer's and Parkinson's disease (2,3). On the other hand, recent studies also suggest that toxicity may precede the formation of large fibrous deposits (3). Phase separation has emerged as an alternative mechanism and has been proposed for several proteins associated to neurodegenerative diseases (4,5), suggesting that protein or peptide molecules with specific sequence properties may self-assemble into oligomeric granular modules with increased toxicity (6). At the same time, amyloid formation also serves as a natural scaffold for the formation of molecular superstructures with impressive functional, protective or structural properties, both in humans and other organisms (7). Amyloid aggregation propensity is encoded in the primary structure of protein molecules, hidden within harboured short sequence segments (8–10). These aggregation prone stretches mediate self-assembly of proteins into ordered perpetuating intermolecular β-sheet assemblies known as ‘cross-β’ spines, which protrude in parallel orientation to the amyloid fibre axis (11). This conformation comprises an extensive network of backbone hydrogen bonds and a set of laterally inter-fitted side chains excluding water molecules, yielding, thus, a tightly packed and energetically favourable amyloid fibril core (12). Aggregation prone regions are usually integral parts buried within the hydrophobic core of the protein native fold and consequently are often enriched with residues favouring β-strand formation, increased hydrophobicity and low charge content (13). Considering such sequence propensities, several computational tools have been developed over the years in an effort to accurately predict aggregation potential from polypeptide sequences (8,14–16). This increasing interest has manifested to a considerable growth in experimental data regarding protein self-assembly regions. Putative aggregation mechanisms based on the notion of aggregation hot spots have been proposed for proteins associated to the formation of both functional and disease-associated amyloid fibrils (17–19). Furthermore, synthetic peptide analogues have been developed as novel strategies for the production of antibacterial or anti-tumoural agents (20,21), for the development of transgenic plants with growth phenotypes (22), or as a new source for the development of potent nanomaterials with various applications (23,24). Following this demand, here we describe the fully updated and significantly expanded WALTZ-DB 2.0, the largest publicly available repository for experimentally determined amyloid-forming peptide sequences.

## EXPANDED CONTENT AND FEATURE IMPROVEMENTS

### New peptide entries and database statistics

WALTZ-DB 2.0 is currently updated to store 1416 hexapeptide entries, divided into nine distinct subsets of origin. In total, 512 peptides have experimentally determined amyloid-forming properties, whereas 904 peptides self-assemble into aggregates with amorphous morphological characteristics. Two novel peptide subsets were added during this update, containing individual peptide mutation screens of known aggregation prone stretches derived from tau and apolipoprotein A-I, which are known amyloid-forming proteins associated to neurodegeneration (25) and atherosclerosis (26). The subsets, designated as ***tau mutant set*** and ***apoAI mutant set***, are composed of 114 and 115 hexapeptides, respectively, and were systematically developed following a single mutation strategy along all available residue positions. Hexapeptide additions were classified into the database as amyloid or non-amyloid-forming sequences when corresponding morphologies were identified, using electron microscopy or by producing positive Thioflavin-T (Th-T) binding spectra. In detail, to characterize a peptide as amyloidogenic, we followed the general convention for amyloid-like morphology. Amyloid fibrils are typically long and unbranched with a diameter ranging between 8 and 10 nm and often tend to coalesce laterally forming superhelices or proto-fibrillar ribbons (27). Once bound to the surface of a β-rich amyloid fibril structure, the benzothiazole Th-T dye displays enhanced fluorescence intensity (28). Peptides producing spectra with increased fluorescence maxima at 480 nm were also considered as amyloid-forming sequences. Following the example of the previous version of the database, all experimental data are available online in order to allow users to independently conclude on the amyloid classification of every hexapeptide entry. Complementary to the above, WALTZ-DB 2.0 also includes an additional number of 98 hexapeptides with detailed annotated amyloid-forming properties which were mined from literature, manually curated and added to the previous *Literature* subset of the database. To summarize, this major update included the storage of 327 new peptide entries, out of which 268 hexapeptides were classified as amyloid-forming and 59 were judged as non-amyloidogenic sequences. Peptide data entries remain stored in a MySQL database available through a web server built with the Drupal content management system. This provides the required infrastructure to keep WALTZ-DB 2.0 regularly up to date, as well as to ensure fast and secure access to the stored data. Finally, a refreshed version of the methods is mentioned on the help page of the website.

### WALTZ-DB 2.0 novel features

**Figure 1.**
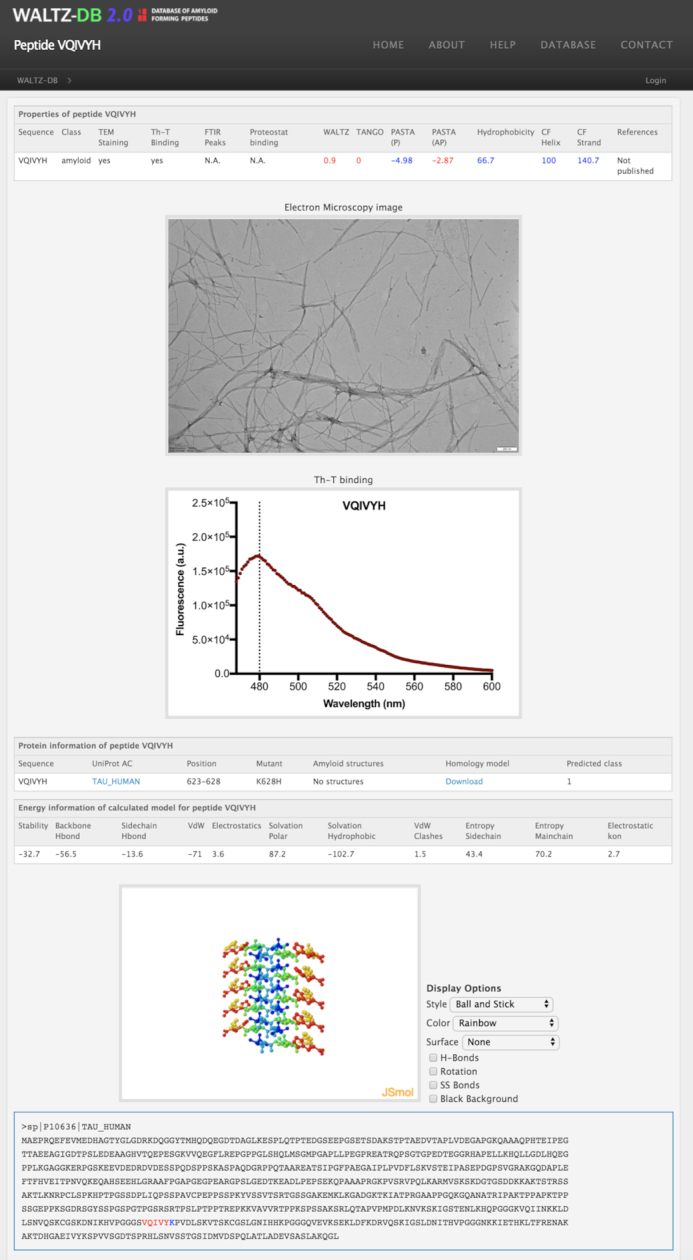
Example output of a peptide entry page on WALTZ-DB 2.0. An upper table contains information on the specific peptide sequence and corresponding predicted propensities. Negative predictions are shown in red, whereas positives are indicated in blue. Electron micrographs and Th-T binding spectra are available online for all new peptide entries. A mutation field indicating the position and transitional mutation for peptide entries has been added in the protein information table, along with a field highlighting the steric zipper class for the model prediction. A new table has been added including detailed energy contributions and the overall stability for the structural model. Finally, a JSmol JmolApplet is used to provide an integrated molecular graphics interface displaying the 3D-structural models of the corresponding hexapeptide entries.

For clarity and in an effort to assist non-experienced users with the evaluation of scoring aggregation properties for peptide entries, predicted aggregation propensities in WALTZ-DB 2.0 are highlighted by specific colouring schemes (Figure 1). In detail, TANGO and WALTZ predictions are shown with a red-to-blue colouring gradient, using thresholds that have been previously reported to provide high specificity (8,15). For sequence hydrophobicity, secondary structure and parallel or antiparallel β-strand formation propensities, positive predictions are shown in blue and negatives in red, respectively. Detailed information on the corresponding threshold values for every individual field can be retrieved from the help page available online.

Every peptide entry stored in the expanded WALTZ-DB 2.0 now contains detailed structural information regarding the putative amyloid fibril core. Utilizing a structural prediction methodology for amyloidogenic sequences developed in our lab, we have analysed all 1416 peptide entries and now provide a structural steric zipper prediction model. Users can download the corresponding models in a PDB format through links that are accessible in the peptide entry pages. Alternatively, WALTZ-DB 2.0 also provides a novel molecular graphics interface panel that is accessible online on every peptide page. This new feature allows users to actively manipulate and investigate peptide model structures on the spot, whilst browsing the online database. The JSmol plugin interface provides several options available for style effects (cartoon, ball and stick, ribbon etc), structural colouring (by secondary structure, residue, element etc) and surface or cavity representation, selection for hydrogen bond or disulphide bridge annotations, as well as modules for structural rotation or background colouring. Adding to this, following structural analysis of the stability of the models and manual curation, all peptide page entries contain information on the energy contributions for the steric zipper models. Energies of all major interactions, such as the contribution of electrostatics, hydrogen bond networking between backbone groups or side chains, solvation energies of residues with hydrophobic or polar properties, as well as Van der Waals packing interactions or potential clashes are attributed to each model entry.

The WALTZ-DB 2.0 database page lists a paged updated table and new filtering steps aiming to make browsing a more user-friendly process. Building on the options of the previous version, the database now offers selections for filtering peptide entries based on availability of Th-T spectral data, as well as energetic stability of the structural models. Using this feature, users can now filter the database and isolate individual sequences by searching within a specific range of overall structural stability energies for predicted steric zippers. Notably, a major disadvantage of the previous version was that users could only download the entire database locally. WATLZ-DB 2.0 now allows users to create and access specific entry datasets by combining any of the provided filters and subsequently downloading the resulting list in a CSV, Excel or JSON format, using buttons that are available at the bottom of the listed table.

## MATERIALS AND METHODS

### Peptide synthesis

Hexapeptides of the new subsets were synthesized using an in-house Intavis Multipep RSi solid phase peptide synthesis robot capable of parallel synthesis of 24–384 peptides. RP-HPLC purification protocols were used to ensure high levels of peptide purification (>90%). Peptide stock solutions were prepared by dissolving in milli-Q water to a final concentration of 1 mM. Dimethyl Sulfoxide (DMSO) traces (<5%) were used to assist with peptide solubility. The peptide solutions were incubated for 2 weeks at 25°C with shaking prior to analysis of amyloid-forming properties.

### Determination of amyloid fibril properties

Transmission electron microscopy was performed to track the morphological properties of the peptide aggregates. Suspensions (5 μl) of peptide aliquots were adsorbed for 1 min to formvar film coated 400-mesh copper grids (Agar Scientific Ltd., England), following a short glow discharge step to improve adsorption. Grids were subsequently washed in 50 μl of milli-Q water and stained with uranyl acetate (2% w/v) for 60 s. Excess stain was removed by blotting with a filter paper. The grids were examined using a JEM-1400 120 kV transmission electron microscope (JEOL, Japan) operated at 80 keV. Amyloid formation was also monitored using Thioflavin-T binding assays. Thioflavin-T (Th-T) is a rotor dye that acts as an efficient reporter of amyloid fibril formation, since it increases its fluorescence when binding to cross-β rich aggregates (28). Th-T (Sigma) was added in low volume black 384-well microplates at a final concentration of 20 μM. Peptide concentration was set to 30 μM. Fluorescence intensity was measured in triplicates, through a ClarioStar plate reader (BMG Labtech, Germany), using an excitation filter at 440 nm and by recording an emission spectrum ranging between 468 and 600 nm. Emission spectra were corrected by subtracting Thioflavin-T - only spectra as background and binding was evaluated by measuring the intensity peak emitted at 480 nm.

### Structural models and energy calculations

To provide a structural characterization for the database peptide entries, we have followed the structural topologies of steric zippers introduced by the Eisenberg lab (10,12,29). Representative 3D-model structures were generated utilizing a structural prediction methodology, developed by the Switch lab. Briefly, this pipeline comprises a large dataset of steric zipper hexapeptide fragment templates that have been extracted from the Protein Data Bank (30). Following implementation of the FoldX energy force field (31), hexapeptide sequences are threaded against all templates, stability energies are calculated and subsequently fed into a random forest classifier. This non-linear classifier then provides a probability estimation of aggregation propensity as a non-trivial function of the corresponding input energies. The threaded structure producing the optimal stability predicted is finally selected as a putative 3D-model representation of the amyloid fibril core.

## LINKS TO OTHER DATABASES

Every WALTZ-DB 2.0 peptide entry is linked to a Uniprot ID (http://www.uniprot.org/) when information of the parental protein is available (32). For peptide entries directly mined from literature, a corresponding reference link connecting to the PUBMED literature portal (http://www.ncbi.nlm.nih.gov/pubmed/) is maintained. Finally, we also provide useful links to other related databases and web servers on protein aggregation (14,29).

## SUMMARY

The former release of WALTZ-DB has served as the largest available repository for amyloid-forming short sequence stretches containing experimental annotation (33). It has been used extensively for the development or as a carefully annotated validation set of several high performing predictors of aggregation propensity (15,16,34–40) and has also been utilized as major source of information incorporated in related databases of amyloid aggregation, such as CPAD (41), AmyLoad (42) and AmyPro (43). Following the above, in the current release we have opted to significantly expand the content of the database by simultaneously doubling the coverage of amyloid-forming peptide sequences (512 amyloid sequences compared to 244 previously available), thus providing an improved and more balanced dataset of entries. Furthermore, structural data and novel online features have been added to promote online access to WALTZ-DB 2.0 as a more user-friendly experience and to provide a new layer of information to the users. Finally, we encourage users to help us keep the database up to date by submitting newly identified aggregation-prone hexapeptide sequences using the contact form available online (http://waltzdb.switchlab.org/contact).
